# Structured noise champagne: an empirical Bayesian algorithm for electromagnetic brain imaging with structured noise

**DOI:** 10.3389/fnhum.2025.1386275

**Published:** 2025-04-07

**Authors:** Sanjay Ghosh, Chang Cai, Ali Hashemi, Yijing Gao, Stefan Haufe, Kensuke Sekihara, Ashish Raj, Srikantan S. Nagarajan

**Affiliations:** ^1^Biomagetic Imaging Laboratory, University of California San Francisco, Department of Radiology and Biomedical Imaging, San Francisco, CA, United States; ^2^Department of Electrical Engineering, Indian Institute of Technology Kharagpur, Kharagpur, India; ^3^National Engineering Research Center for E-Learning, Central China Normal University, Wuhan, China; ^4^Technical University Berlin, Berlin, Germany; ^5^Signal Analysis Inc., Tokyo, Japan

**Keywords:** electromagnetic brain imaging, magnetoencephalography (MEG), brain source reconstruction, Bayesian inference, structured noise learning, factor analysis

## Abstract

**Introduction:**

Electromagnetic brain imaging is the reconstruction of brain activity from non-invasive recordings of electroencephalography (EEG), magnetoencephalography (MEG), and also from invasive ones such as the intracranial recording of electrocorticography (ECoG), intracranial electroencephalography (iEEG), and stereo electroencephalography EEG (sEEG). These modalities are widely used techniques to study the function of the human brain. Efficient reconstruction of electrophysiological activity of neurons in the brain from EEG/MEG measurements is important for neuroscience research and clinical applications. An enduring challenge in this field is the accurate inference of brain signals of interest while accounting for all sources of noise that contribute to the sensor measurements. The statistical characteristic of the noise plays a crucial role in the success of the brain source recovery process, which can be formulated as a sparse regression problem.

**Method:**

In this study, we assume that the dominant environment and biological sources of noise that have high spatial correlations in the sensors can be expressed as a structured noise model based on the variational Bayesian factor analysis. To the best of our knowledge, no existing algorithm has addressed the brain source estimation problem with such structured noise. We propose to apply a robust empirical Bayesian framework for iteratively estimating the brain source activity and the statistics of the structured noise. In particular, we perform inference of the variational Bayesian factor analysis (VBFA) noise model iteratively in conjunction with source reconstruction.

**Results:**

To demonstrate the effectiveness of the proposed algorithm, we perform experiments on both simulated and real datasets. Our algorithm achieves superior performance as compared to several existing benchmark algorithms.

**Discussion:**

A key aspect of our algorithm is that we do not require any additional baseline measurements to estimate the noise covariance from the sensor data under scenarios such as resting state analysis, and other use cases wherein a noise or artifactual source occurs only in the active period but not in the baseline period (e.g., neuro-modulatory stimulation artifacts and speech movements).

## 1 Introduction

Electromagnetic brain imaging is an effective technique being used intensively to understand the neural mechanisms of the complex human brain and behavior important for both neuroscience research and clinical applications (Phillips et al., 1997; He et al., 2018). In particular, electroencephalography (EEG) and magnetoencephalography (MEG) are two widely used techniques that provide non-invasive recordings of the electrical activity of the brain by sensing its remote magnetic and electric fields, respectively (Baillet et al., 2001). Electromagnetic brain imaging requires solving an ill-posed inverse problem for reconstruction of brain activity (at cortical brain sources) from non-invasive EEG/MEG recordings. In particular, it is crucial to determine both the spatial location and the temporal dynamics of neurophysiological activity. In tomographic EEG/MEG source localization pipelines, current dipole sources are considered to be located on each voxel inside the brain. As a result, the number of locations of potential brain sources (thousands of voxels) is typically much larger than the number of sensors (just a few hundred). In addition, several types of noise (such as environmental interference and sensor noise) inevitably affect EEG/MEG signals (Michel and He, 2019; Edelman et al., 2015). Therefore, reconstructing brain source activities accurately from scalp EEG/MEG measurements becomes a challenging task. It opens up the possibility of using sophisticated mathematical or neurophysiological priors of both brain signals and noise statistics to achieve improved recovery of brain activities.

Integral to inverse modeling algorithm in electromagnetic brain imaging (Riaz et al., 2023) are forward models. The primary current density is mathematically defined as a *vector field* in a continuum (volume space or surface space, depending on the assumptions for the source space). Then an electric scalar field or magnetic vector field that is produced by the primary current density is described by quasi-static equations of electromagnetism. A discretization of the space of the brain and surrounding tissues is employed to provide these equations with a numerical solution. Note that the forward problem aims to achieve a solution for the electric or magnetic fields given the location and timing of brain source activity. The recovery of the primary current source location and timing is basically the Cauchy inverse problem of electromagnetism (Riaz et al., 2023).

Several methods have been introduced over the past decades to solve the inverse problem of brain source imaging. Common inverse solvers for EEG/MEG source imaging can be broadly classified into three categories—model-based dipole fitting, dipole scanning methods, and distributed whole brain source imaging methods (Baillet et al., 2001; Cai et al., 2018; Hosseini et al., 2018; Cai et al., 2023). The working principle of dipole fitting methods is to approximate brain activity with a small number of equivalent current dipoles (Scherg, 1990). These classical methods achieve good solutions when the source activity is relatively simple consisting only one to two dipoles In addition, it is practically challenging for dipole fitting-based methods to determine the true number of current dipoles to be estimated. Dipole scanning methods are also referred to as methods of spatial filtering or beamforming which estimate the time course at every candidate location while suppressing the interference from activity at the other candidate source locations (Van Veen et al., 1997; Zumer et al., 2006; Cai et al., 2023). Examples of scanning techniques are minimum-variance adaptive beamforming (Robinson and Rose, 1992; Sekihara and Scholz, 1996) as well as several variants of adaptive beamformers (Sekihara and Nagarajan, 2008). The fidelity of such brain signal estimates is affected by many factors such as signal-to-noise ratio (SNR), source correlations, and the number of time samples. However, the reconstruction performance of beamforming methods can be significantly compromised if the brain sources are highly correlated, although recent Bayesian extensions overcome this limitation (Cai et al., 2023).

Distributed whole-brain source imaging methods do not require prior knowledge of the number of sources (Wipf and Nagarajan, 2009). These methods approximate the primary electrical current density by discretizing the whole brain volume, assuming a dipolar current source at each voxel. The task is then to estimate the amplitudes (and orientations) of the sources by minimizing a cost function (He et al., 2011). Some form of prior constraints or regularizers are used to obtain a unique and neurophysiological meaningful solution (Ioannides et al., 1990). The minimum-norm estimation algorithm (MNE) (Hämäläinen and Ilmoniemi, 1994) minimizes the *L*_2_ norm of the solution favoring smaller overall power of the brain activity. Other variants of MNE include the weighted MNE (wMNE) (Dale and Sereno, 1993), low-resolution brain electromagnetic tomography (LORETA) (Pascual-Marqui et al., 1994), and standardized LORETA (sLORETA) (Pascual-Marqui et al., 2002). However, *L*_2_ norm minimization methods produce diffuse estimates that lack sufficient resolution to localize and distinguish multiple sources. To overcome this limitation, algorithms based on *L*_1_ norm minimization (Ding and He, 2008; Liu et al., 2022) and sparsity-inducing norms induced by empirical Bayesian inference (referred to as sparse Bayesian learning, SBL) (Wipf et al., 2010) are developed. Friston et al. (2008) introduced a sparse solution for distributed sources, of the sort enforced by equivalent current dipole (ECD) models. These sparsity-based source reconstruction algorithms can be derived within a Bayesian framework (Wipf et al., 2010; Liu et al., 2019; Oikonomou and Kompatsiaris, 2020; Liu et al., 2020; Cai et al., 2021; Hashemi et al., 2021b, 2022; Ghosh et al., 2023; Cai et al., 2023). We argue that these Bayesian techniques are found to be most efficient in estimating the model hyper-parameters directly from the data using hierarchical algorithms. Importantly, the Champagne algorithm (Wipf et al., 2010) is derived in an empirical Bayesian fashion, incorporating deep theoretical ideas about sparse-source recovery from noisy constrained measurements. Inspired by its promising performance, attempts have further been made by several researchers to improve upon Champagne algorithm (Wipf et al., 2010). One potential direction of improvement is to accurately model noise that exhibits structured precision parameter (Liu et al., 2020).

Accurate inference of brain signals of interest while accounting for all sources of noise that contribute to sensor measurements is the key challenge in electromagnetic brain imaging. Noise statistics in the model play a crucial role in the success of sparse source recovery. In particular, the statistical characteristics of the noise in sensor data plays an important role in the working of Bayesian algorithms for electromagnetic brain imaging. Existing studies have considered noise covariance matrices with either diagonal (Wipf et al., 2010; Cai et al., 2019) or full structure (Hashemi et al., 2022). In this study, we consider another type of realistic noise whose covariance can be characterized by a structured matrix. This type of noise is present when there are just a few active sources of environmental noise, each of which may be picked up by multiple MEG/EEG sensors with high spatial correlations. A key aspect of our noise estimation algorithm is that we do not require any additional baseline measurements to estimate the noise covariance from the sensor data under scenarios such as resting-state analysis, and other use cases wherein a noise or artifactual source occurs only in the active period but not in the baseline period (e.g., neuromodulatory stimulation artifacts and speech movements). To the best of our knowledge, no existing algorithm has addressed the brain source estimation problem with a structure-noise covariance.

The main contributions of this paper are as follows:

1. We introduce a novel robust empirical Bayesian framework for electromagnetic brain imaging under the structured noise covariance assumption. In particular, we perform inference of the variational Bayesian factor analysis (VBFA) noise model iteratively in conjunction with source reconstruction. It provides us a tractable algorithm for iteratively estimating the noise covariance and the brain source activity. The proposed algorithm is found to be quite robust to initialization and computationally efficient.
2. The proposed algorithm does not require any additional baseline measurements to estimate noise covariance from sensor data. We note that this is not the case for many of the existing algorithms for electromagnetic brain imaging.
3. We perform exhaustive experiments to demonstrate the effectiveness of the proposed electromagnetic brain imaging algorithm on both simulated and real datasets. In particular, we quantify the correctness of the localization of the sources and the estimation of source time courses for simulated brain noise with structured covariance matrix. We show that the new algorithm achieves competitive performance with respect to benchmark methods on both synthetic and real MEG data and is able to resolve distinct and functionally relevant brain areas.

This paper is summarized as follows. In Section 2.1, we introduce generative model of the inverse problem. Table 1 list a summary of the variables and definitions used in Section 2. The proposed Bayesian formulation along with a brief account of existing Bayesian frameworks are presented in Section 2.2 and Section 2.3. Then, we present experiments applying our approach on synthetic (Section 3) and real MEG data (Section 4), where we also compare the proposed algorithm with baseline and state-of-the-art methods for electromagnetic brain imaging. Finally, we discuss implications and future directions in Section 5.

**Table 1:** Summary of the variables and definitions used in Section 2 Method.

**Symbol**	**Description**
*n*	Number of voxels in the brain.
*m*	Number of MEG/EEG sensors.
*K*	Number of time-points in the MEG/EEG signal.
***x***	Brain source signal. (size *n* × *K*)
***y***	Observed MEG/EEG sensor data. (size *m* × *K*)
***L***	Lead-field matrix of size *m* × *n*.
**Λ**	Precision of normal distribution of noise. (size *m* × *m*)
**Φ**	Precision of normal distribution of source signal. (size *n* × *n*)
**Γ**	Precision of normal posterior distribution of source signal *x* given observed data *y*. (size *n* × *n*)
*p*(***x***_*k*_)	Prior distribution of ***x***_*k*_.
*p*(***y***_*k*_|***x***_*k*_)	Conditional probability density of ***y***_*k*_ given ***x***_*k*_.
*p*(***x***_*k*_|***y***_*k*_)	Posterior probability density of ***x***_*k*_ observed ***y***_*k*_.
${\bar{x}}_{k}$	Posterior mean of *p*(***x***_*k*_|***y***_*k*_).
*p*(***y***|**Φ**)	Marginal likelihood.
F(**Φ**)	Logarithm of marginal likelihood log*p*(***y***|**Φ**).
$\tilde{F}\left(\Phi \right)$	Cost function with the convex bounding.
$z_{k}^{\left(l\right)}$	Residual noise at *k*-th time-point during *l*-th iteration. (size *m* × 1)
***A***	Mixing matrix for the factor analysis. (size *m* × *q*)
***u***_*k*_	k-th component of factor analysis. (size *q* × 1)
ε	Modeling noise of factor analysis. (size *m* × 1)
**Ω**	Diagonal precision matrix of the modeling noise at factor analysis step.
**Ψ**	Precision matrix of the posterior distribution of column of mixing matrix ***A***.
***R***_*zz*_	Covariance of residual noise ***z***.
***R***_*uu*_	Covariance of factor analysis coefficient vector ***u***.

## 2 Method

### 2.1 Generative model

In the typical electromagnetic brain imaging problem setup, brain activity is modeled by a number of electric current dipoles, where the location, orientation, and magnitude of each dipole collectively determine the signal observed at the EEG/MEG electrodes. The position of the dipoles within the brain contains valuable information on brain function, which is used in clinical applications and cognitive neuroscience studies (Leahy et al., 1998; Gross, 2019). The inverse problem of estimating the locations and the moments of the current dipoles from the recorded EEG signal is ill-posed in nature.

The forward model, describing the EEG/MEG measurements as a function of the brain sources, is given by


(1) $$\begin{array}{l} y_{k}=Lx_{k}+z_{k}, \end{array}$$

where $y_{k}={\left[y_{k}\left(1\right),\cdots \;,y_{k}\left(m\right)\right]}^{⊤},y_{k}\in {\mathbb{R}}^{m\times 1}$ is the sensor measurements at time point *k*, *m* is the number of sensor measurements. Moreover, $x_{k}={\left[x_{k}\left(1\right),\cdots \;,x_{k}\left(n\right)\right]}^{⊤}$, $x_{k}\in {\mathbb{R}}^{n\times 1}$ is the activity of the brain sources at time point *k*, *n* is the number of voxels. In addition, the whole time series data {***y***_1_, ***y***_2_, …, ***y***_*K*_} are collectively denoted ***y***, and the whole time series data {***x***_1_, ***x***_2_, …, ***x***_*K*_} are collectively denoted ***x***. The lead-field matrix is given by $L=\left[l_{1},l_{2},\cdots \;,l_{n}\right]\in {\mathbb{R}}^{m\times n}$ whose columns reflect the sensors response induced by the unit current sources. Note that, in simulations here, we assume a pre-defined orientation (e.g., normal constraint) for the local lead-field at each voxel. Therefore, it can be reduced to a *m* × 1 vector (Sekihara and Nagarajan, 2015). However for real data, we use a three-column lead-field for each voxel and estimate the source time-series at the orientation corresponding to maximum power at each vowel. Furthermore, $z_{k}\in {\mathbb{R}}^{m\times 1}$ refers to additive noise in the measurements not arising from brain sources. We consider that ***z***_*k*_ is drawn from a multivariate Gaussian probability distribution parameterized by the precision matrix **Λ**^−1^. In particular, we assume that noise refers to any background interference including biological and environmental sources outside the span of the lead-field as well as sensor noise. It is assumed that EEG/MEG measurements are collected for spontaneous brain activity (i.e., resting-state), such that separate recording time-windows capturing noise only activity might not be available. Another scenario where noise-only recordings may not be available are task-induced contrastive experimental designs where noise or artifact signals are only present in the active condition but not in the baseline. One such example is active post-movement related paradigms such as speaking or other movement tasks wherein any artifacts observed in the sensors due to the movement will be present in the post-movement period; that is, the baseline pre-movement periods cannot be used to estimate the noise statistics. Therefore, here, we jointly infer both source estimate and noise statistics from the same data segment as described below.

### 2.2 Source estimation

Given prior distributions of sources and noise, the generative model in Equation 1 becomes a probabilistic model. We assume a zero-mean Gaussian prior with diagonal covariance **Φ** = diag(**ϕ**) for the underlying source distribution. In other words, $x_{k}\sim N\left(0,\Phi^{-1}\right),\;k=1,\ldots ,K$, where the diagonal $\phi ={\left[\phi_{1},\ldots ,\phi_{n}\right]}^{⊤}$ contains *n* distinct unknown variances associated with *n* brain sources.

We propose to solve the inverse problem within the Bayesian learning framework. Modeling independent sources through a Gaussian zero-mean prior with diagonal covariance matrix leads to sparsity of the resulting source distributions, that is, at the optimum many of the estimated source variances are zero. The goal is to find the maximum a posteriori probability (MAP) solution for *x*_*k*_. The posterior probability *p*(***x***_*k*_|***y***_*k*_) can be derived by using Bayes' theorem (Sekihara and Nagarajan, 2015):


(2) $$\begin{array}{l} p\left(x_{k}|y_{k}\right)\propto p\left(y_{k}|x_{k}\right)p\left(x_{k}\right), \end{array}$$

where $p\left(y_{k}|x_{k}\right)=N\left(Lx_{k},\Lambda^{-1}\right)$ and $p\left(x_{k}\right)=N\left(0,\Phi^{-1}\right)$. In this case, it is straightforward to show that the posterior probability density *p*(***x***_*k*_|***y***_*k*_) is also Gaussian. Suppose, the posterior probability takes the following form:


$$\begin{array}{l} p\left(x_{k}|y_{k}\right)=N\left({\bar{x}}_{k},\Gamma^{-1}\right), \end{array}$$

where ${\bar{x}}_{k}$ is the posterior mean, and **Γ** is the posterior precision matrix. Furthermore, we adapt the derivation in Sekihara and Nagarajan (2015)[See pages 233–235 in Section B.3] to obtain:


(3) $$\begin{array}{l} \begin{array}{c} \Gamma =\Phi +L^{T}\Lambda L,\\ {\bar{x}}_{k}=\Phi^{-1}L^{⊤}{\left(\Lambda^{-1}+L\Phi^{-1}L^{⊤}\right)}^{-1}y_{k}. \end{array} \end{array}$$

Note that we need both **Φ** and **Λ** to compute ${\bar{x}}_{k}$. Assuming **Λ** and **Φ** as follows are known, we repeat the following three iterative steps until convergence. At the (*l* + 1)-th iteration:

1. Estimate ${\bar{x}}_{k}^{\left(l+1\right)}$, assuming known **Λ**^(*l*)^ and **Φ**^(*l*)^.
2. Estimate **Φ**^(*l*+1)^, assuming known ${\bar{x}}_{k}^{\left(l+1\right)}$ and **Λ**^(*l*)^.
3. Estimate **Λ**^(*l*+1)^, assuming known ${\bar{x}}_{k}^{\left(l+1\right)}$ and **Φ**^(*l*+1)^.

We estimate **Φ** in the (*l* + 1)-th iteration by maximizing the following cost function, which is defined as the logarithm of marginal likelihood *p*(***y***|**Φ**) given **Φ**:


(4) $$\begin{array}{l} \begin{array}{c} F\left(\Phi \right)=\log|\Sigma_{y}|+\frac{1}{k}\overset{K}{\underset{k=1}{\sum }}y_{k}^{⊤}\Sigma_{y}^{-1}y_{k}, \end{array} \end{array}$$

where the model data covariance


(5) $$\begin{array}{l} \begin{array}{c} \Sigma_{y}=\Lambda^{-1}+L\Phi^{-1}L^{⊤}. \end{array} \end{array}$$

Then, similar to Champagne (Wipf et al., 2010; Cai et al., 2019), we utilize a convex bounding on the cost logarithm (Equation 4),


(6) $$\begin{array}{l} \begin{array}{c} \tilde{F}\left(\Phi \right)=\frac{1}{K}\overset{K}{\underset{k=1}{\sum }}\left[{\left(y_{k}-L{\bar{x}}_{k}\right)}^{⊤}\Lambda^{-1}\left(y_{k}-L{\bar{x}}_{k}\right)\right]\\ +\frac{1}{K}\overset{K}{\underset{k=1}{\sum }}{\bar{x}}_{k}^{⊤}\Phi^{-1}{\bar{x}}_{k}+\text{tr}\left(g^{⊤}\Phi \right)+g_{0}, \end{array} \end{array}$$

where ***g*** = diag(*g*_1_, *g*_2_, ⋯ , *g*_*n*_) and *g*_0_ are auxiliary variables. Setting the derivative of $\tilde{F}\left(\Phi \right)$ with respect to ϕ_*i*_ and *g*_*i*_ generates the update rules below,


(7) $$\begin{array}{l} \begin{array}{c} \hat{\phi_{i}}=\sqrt{\frac{\frac{1}{K}\sum_{k=1}^{K}{\bar{x}}_{k}^{2}\left(i\right)}{\hat{g_{i}}}},\\ \hat{g_{i}}=l_{i}^{⊤}\Sigma_{y}^{-1}l_{i}. \end{array} \end{array}$$

The update rule of **Φ** is defined as $\hat{\Phi }=diag\left({\hat{\phi }}_{1},{\hat{\phi }}_{2},\cdots \;,{\hat{\phi }}_{n}\right)$.

### 2.3 Structured noise estimation using variational Bayesian factor analysis

To estimate **Λ**, we perform inference on a variational Bayesian factor analysis model of the following residual noise at the *l*-th iteration (Nagarajan et al., 2007):


(8) $$\begin{array}{l} z_{k}=y_{k}-L{\bar{x}}_{k}, \end{array}$$

where the residual noise at the *k*-th time-point is $z_{k}\in {\mathbb{R}}^{m\times 1}$. We note that our estimation problem involving a multivariate Gaussian noise process becomes intractable if both the source covariance is non-diagonal and non-sparse and the noise covariance is full rank. The assumption of structured noise helps with accurate estimation with the use of the variational Bayesian factor analysis methods which are robust to smaller data sizes and to the underlying factor dimension specification. The joint estimation of both diagonal sparse source covariance and structured noise covariance is what we are aiming for. Currently, we perform factor analysis-based decomposition of ***x***_*k*_ as follows:


$$\begin{array}{l} z_{k}=Au_{k}+\varepsilon , \end{array}$$

where ***A*** ∈ ℝ^*m*×*q*^ is a mixing matrix, ***u***_*k*_ is a *q*-dimensional column vector, and **ε** is modeling noise. Notice that we drop the iteration symbol *l* for simplification of notations.

We further assume the prior probability distribution of the factor *u*_*k*_ to be the zero-mean Gaussian with its precision matrix equal to the identity matrix as follows:


$$\begin{array}{l} p\left(u_{k}\right)=N\left(0,\text{I}\right). \end{array}$$

We define the *j*-th row of mixing matrix *A* as a column vector ***a***_*j*_ such that


$$\begin{array}{l} A=\left[\begin{array}{c} a_{1}^{T}\\ a_{2}^{T}\\ \vdots \\ a_{M}^{T} \end{array}\right]. \end{array}$$

Currently we assume the prior distribution of ***a***_*j*_ to be:


(9) $$\begin{array}{l} p\left(a_{j}\right)=N(0,{\left(\sigma_{j}\alpha \right)}^{-1}), \end{array}$$

where the *j*-th diagonal element of the modeling noise precision matrix Σ is denoted σ_*j*_, and **α** = diag(α_1_, α_2_, …, α_*q*_) is a diagonal matrix. It can be further shown that the posterior probability distribution has the form of the Gaussian distribution:


$$\begin{array}{l} p\left(a_{j}|z\right)=N({\bar{a}}_{j},{\left(\sigma_{j}\text{Ψ}\right)}^{-1}), \end{array}$$

where ***z*** = [***z***_1_, ⋯ , ***z***_*K*_] is the residual signal of *K* time points, and ${\bar{a}}_{j}$ and σ_*j*_**Ψ** are the mean and precision matrix of the posterior distribution.

For simplicity, the prior probability distribution of the factor ***u***_*k*_ is assumed to be the zero-mean Gaussian with its precision matrix equal to the identity matrix,


$$\begin{array}{l} p\left(u_{k}\right)=N\left(0,\text{I}\right). \end{array}$$

The factor activity ***u*** = [***u***_1_, ⋯ , ***u***_*K*_] is assumed to be independent across time. Thus, the joint prior distribution has the form


$$\begin{array}{l} p\left(u\right)=\overset{K}{\underset{k=1}{\prod }}p\left(u_{k}\right)=\overset{K}{\underset{k=1}{\prod }}N\left(0,\text{I}\right), \end{array}$$

where **I** is an identity matrix of size *P* × *P*. The modeling noise ε is assumed to be Gaussian with the mean of zero:


$$\begin{array}{l} p\left(\varepsilon \right)=N\left(0,\Omega^{-1}\right), \end{array}$$

where **Ω** is a diagonal precision matrix. Currently, one can show that the posterior distribution *p*(***u***_*k*_|*z*_*k*_) is also Gaussian, which we assume to be:


$$\begin{array}{l} p\left(u_{k}|z_{k}\right)=N\left({\bar{u}}_{k},\Sigma_{u}^{-1}\right), \end{array}$$

where ${\bar{u}}_{k}$ and **Σ**_*u*_ are mean and precision, respectively.

By using the variational Bayesian expectation maximization (EM) algorithm, it can be derived that


(10) $$\begin{array}{l} \Sigma_{u}=A^{⊤}\Omega A+m{\text{Ψ}}^{-1}+\text{I} \end{array}$$

(11) $$\begin{array}{l} {\bar{u}}_{k}=\Sigma_{u}^{-1}A^{⊤}\Omega z_{k} \end{array}$$

(12) $$\begin{array}{l} A=R_{zu}{\left(R_{uu}+\alpha \right)}^{-1} \end{array}$$

(13) $$\begin{array}{l} \text{Ψ}=R_{uu}+\alpha , \end{array}$$

where $R_{uu}=E_{u}[\sum_{k=1}^{K}u_{k}u_{k}^{⊤}]$, $R_{zu}=E_{u}[\sum_{k=1}^{K}z_{k}u_{k}^{T}]$. Note that the hyper-parameters **α** and **Ω** can be updated as follows:


(14) $$\begin{array}{l} \begin{array}{c} \alpha^{-1}=\text{diag }[\frac{1}{m}A^{⊤}\Omega A+{\text{Ψ}}^{-1}]\\ \Omega^{-1}=\frac{1}{K}[R_{zz}-AR_{uz}], \end{array} \end{array}$$

where $R_{zz}=E_{u}[\sum_{k=1}^{K}z_{k}z_{k}^{⊤}]$ and $R_{uz}=R_{zu}^{T}$. Finally, we iteratively update the above equations until the free energy function converged and the covariance matrix of the structured noise computed only using the signal of interest is given by


(15) $$\begin{array}{l} \Lambda =E\left[zz\right]=\frac{1}{K}AR_{uu}A^{⊤}+\frac{1}{K}\Omega^{-1}\text{tr}\left(R_{uu}{\text{Ψ}}^{-1}\right). \end{array}$$

We refer to the proposed brain source imaging method as *structured noise Champagne (SNC)*. The key aspect is the novel way of estimating the covariance of the residual noise within each iteration.

## 3 Simulation experiments

In this section, we focus on experiments with simulated data. In particular, we follow a standard protocol from the literature for simulating MEG source signals. We combine this with simulated structured noise of which the precision matrix has low rank. More details are provided in Section 3.3.

### 3.1 Quantifying performance

The performance of brain source reconstructions is evaluated using response receiver operating characteristics (FROC) (Cai et al., 2021). It basically measures the probability of detecting a true source in an image vs. the expected value of the number of false-positive detection per image. We further compute the *A*′ metric which is the area under the FROC curve (Owen et al., 2012; Snodgrass and Corwin, 1988). Note that the *A*′ metric determines the hit rate (*h*_*r*_) of correctly detecting the active sources. We define the hit rate (*h*_*r*_) as the number of hits for dipolar sources divided by the true number of dipolar sources in the brain. A dipolar source is considered as hit when recovered signal power is beyond a certain threshold value. First, the voxels localized by each algorithm that are included in the calculation of hit rates are defined as voxels that are (i) at least 1% of the maximum activation of the localization result and (ii) within the largest 10% of all of the voxels in the brain. Within these subsets of voxels, we test whether each voxel is within the ten nearest voxels to a true source. If estimated activity of a particular voxel lies within a true source, that source gets labeled as a “hit.” We also define another metric false positive rate (*f*_*r*_) as the number of potential false-positive dipolar sources divided by the total number of false dipolar sources. Note that a larger AOC value indicates a higher hit rate (*h*_*r*_) than a false-positive rate (*f*_*r*_). The expression for the *A*′ metric (Owen et al., 2012) is given by:


(16) $$\begin{array}{l} A^{\prime }=\frac{1}{2}\left(h_{r}+f_{r}\right)+\frac{1}{2}. \end{array}$$

In our experiments, we also study the accuracy of the time course reconstructions. This accuracy metric $\bar{R}$ is defined as the correlation coefficient between the seed and estimated source time courses for each hit. The overall performance of both the accuracy of the localization and reconstruction of time courses is computed by combining *A*′ and $\bar{R}$. The aggregated performance (AP) is given by Cai et al. (2019):


(17) $$\begin{array}{l} AP=\frac{1}{2}\left(A^{\prime }+h_{r}\bar{R}\right). \end{array}$$

We see that *AP* values range in [0, 1]. A higher value of *AP* indicates a better overall performance of source localization and time course reconstruction.

### 3.2 Benchmarking methods

We compare our method *structured noise Champagne (SNC)* with the following existing source localization methods:

1. Minimum current estimate (MCE) (Matsuura and Okabe, 1995),
2. Standardized low-resolution brain electromagnetic tomography (sLORETA) (Pascual-Marqui et al., 2002),
3. Linearly constrained minimum variance (LCMV) (Van Veen et al., 1997),
4. Noise learning Champagne (NLC) (Cai et al., 2021).

We apply these existing methods within the targeted structured noise model. For experiments with real data, we first estimate the noise variance using the variational Bayesian factor analysis (VBFA) algorithm (Nagarajan et al., 2007) and use the original Champagne (Nagarajan et al., 2007) to estimate the location of active brain sources. This result would set an upper bound on the performance of Champagne with noise learning when baseline data are not available for real data.

### 3.3 MEG simulations

We generate source signal data by simulating dipole sources with a fixed orientation. Damped sinusoidal oscillations with frequencies sampled randomly between 1 and 75 Hz are created as voxel source time courses. The time-courses are then projected to the sensors using the lead-field matrix generated by the forward model. We consider 271 MEG sensors and a single-shell spherical model (Hallez et al., 2007) implemented in SPM12 (http://www.fil.ion.ucl.ac.uk/spm) at the default spatial resolution of 8,196 voxels corresponding approximately to a 5-mm inter-voxel spacing. We simulated 480 samples for which sampling frequency is 1200 Hz and signal duration is 0.8 s.

**Figure 1:**
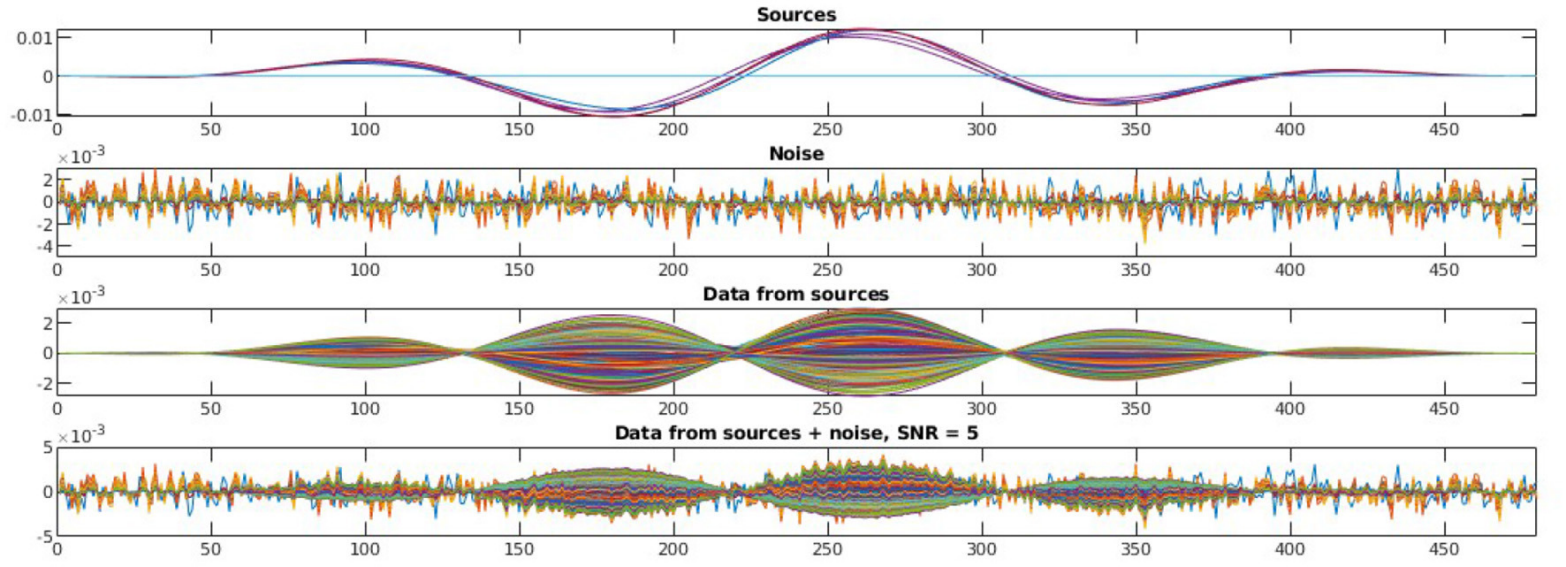
Simulated MEG signal, structured noise, and measurement from MEG sensors. In this experiment, we have five brain sources as we see on the top plot. The second plot shows the noise generated as per our model of structured noise. The third plot presents the measured signal at the MEG sensor resulting only from the active brain source time-series—***Lx*** in Equation 1. Finally, the time-series at the bottom is the one captured at MEG sensors resulting from both active sources and noise—*y* in Equation 1. For all these, we show 480 time-points.

**Figure 2:**
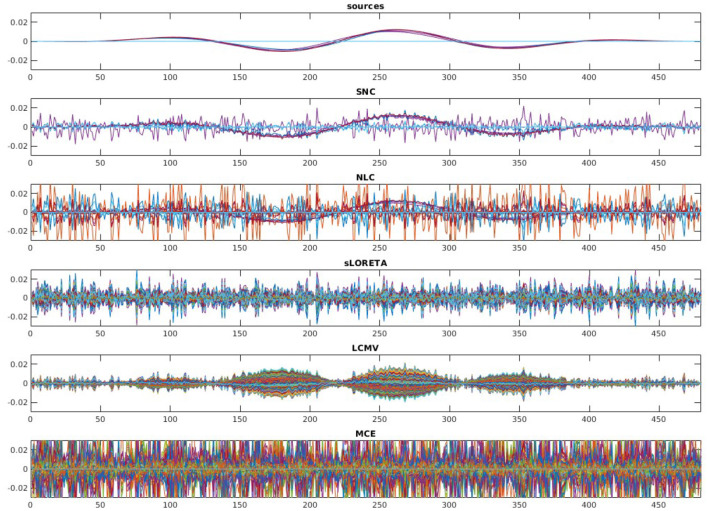
Reconstruction results on the simulated MEG measurement shown in Figure 1. Recall, we have five brain sources in this experiment. It is desirable for a good brain source imaging method to recover the brain source signal successfully while also suppressing the noise. Notice that in this experiment for the considered structured noise model, noise learning Champagne (NLC), and our method structured noise Champagne (SNC) are able to mitigate the noise more successfully. Between them, SNC is able to produce cleaner brain source time-series. In this experiment, standardized low-resolution brain electromagnetic tomograph (sLORETA), linearly constrained minimum variance (LCMV), and minimum current estimate (MCE) failed to achieve satisfactory reconstruction.

**Figure 3:**
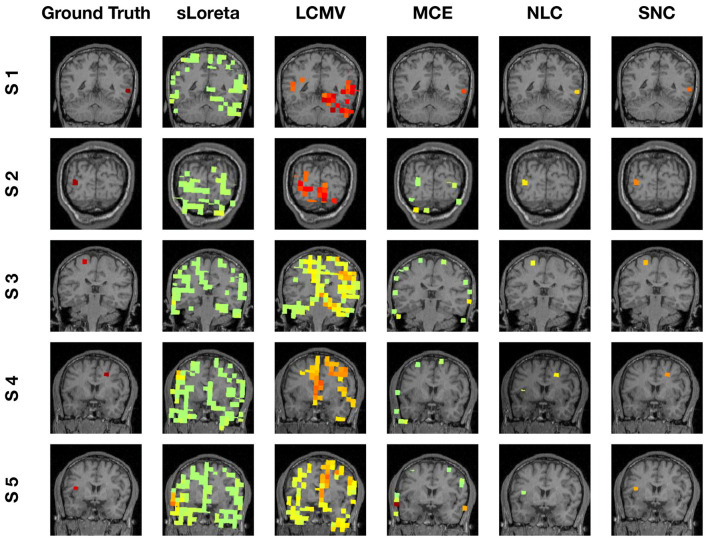
Localization results of simulation experiment (visualization of brain sources power). In this experiment, we simulated five brain sources: S1, S2, S3, S4, and S5. For each brain source, we display the localization by all five methods. Notice that our method SNC is able to localize the sources most accurately.

To evaluate the robustness of the proposed method, we randomly choose noise activity with real brain noise consisting of actual resting-state sensor recordings collected from ten human subjects presumed to have only spontaneous brain activity and sensor noise. Signal-to-noise ratio (SNR) and correlations between voxel time courses are varied to examine algorithm performance. The SNR and time course correlation are defined in Owen et al. (2012). We show an example of time-course reconstruction using our proposed method in Figures 1, 2. The top plot in Figure 1 is the simulated ground-truth MEG signal with five active sources. This is followed by the time-course at the MEG sensor (before adding the noise). Moreover, finally, the time-course at the bottom in Figure 1 is the measured signal at the MEG sensor with additive noise of 5 dB. The reconstructed time-series using our method SNC is shown in Figure 2. We also display the time-series reconstructions obtained using NLC (Cai et al., 2021), sLORETA (Pascual-Marqui et al., 2002), LCMV (Van Veen et al., 1997), and MCE (Matsuura and Okabe, 1995). It is noteworthy that the new method SNC is able to reconstruct the time-series best. For all simulations, the inter-source correlation coefficient was fixed at 0.99 and the SNR was fixed at 3 dB. To highlight the source localization by all five methods, we show the power at each voxel of the reconstructed time-series and compare it with the ground-truth in Figure 3. Notice that our method SNL achieves the best results to rightly localize the brain sources. In other words, it shows the localization performance of the methods in the simulation experiment.

**Figure 4:**
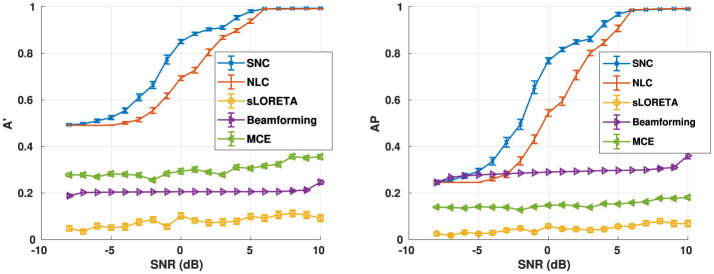
Aggregate performance in simulations for varying noise levels for the number of noise factors *m* = 40. In this experiment, we use five active brain sources in our simulation. On the other hand, we first simulated a structured noise drawn from N(**0**, **Λ**^−1^). Furthermore, we use the knowledge of the structured noise model at the heart of our method to reconstruct the voxel-level time-series. Notice that our method structured noise Champagne (SNC) performs better than every existing method in terms of correctly localizing the active brain sources.

We also evaluate algorithm performance as a function of SNR, as shown in Figure 4. The reconstruction performance is evaluated for five randomly seeded dipolar sources with an inter-source correlation coefficient of 0.99. The simulations were performed at SNRs from –8 dB to 10 dB at a step of 1 dB. Both metrics suggest that our method structured noise Champagne (SNC) is able to localize the active brain sources more accurately than the existing methods.

**Figure 5:**
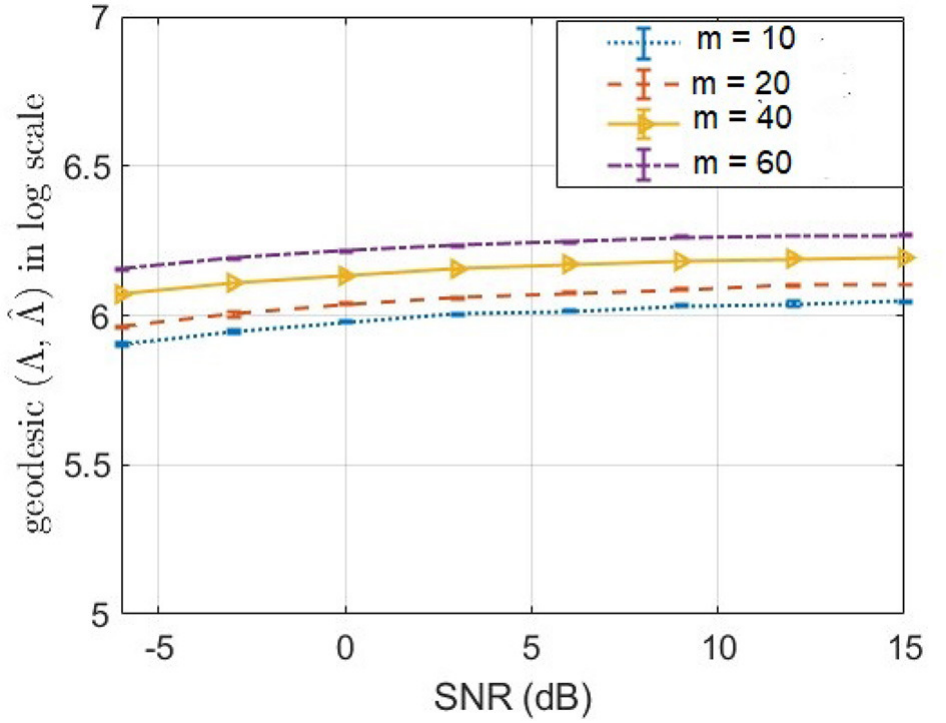
Geodesic distance (Venkatesh et al., 2020) between true and estimated precision matrices of structured noise for a different number of noise factors *m*.

Our method structured noise Champagne (SNC) deviates from the original Champagne algorithm by the way of noise precision update step. In the original Champagne algorithm, Λ is learnt from available baseline or control measurements (Wipf et al., 2010). In contrast, here in structured noise Champagne (SNC), we update rules for estimation of a diagonal noise covariance, without baseline measurements. In Figure 5, we demonstrate how well the structured noise is reconstructed. In particular, we compute the geodesic distance (Venkatesh et al., 2020) between true structured noise and the reconstructed one using SNC. Then, we plot the geodesic distance across a range of signal-to-noise ratio (SNR) for different values of rank. We found that our method SNC is able to predict the structured noise fairly consistently across SNR values. However, we empirically also found that there is still scope for improvement in the estimated noise precision. We will focus on new technical innovations to address this limitation in our future research.

## 4 Analysis on real MEG data

Real MEG data were acquired in the Biomagnetic Imaging Laboratory at the University of California, San Francisco (UCSF) with a CTF Omega 2000 whole-head MEG system from VSM MedTech (Coquitlam, BC, Canada) at a 1,200 Hz sampling rate. Formal consent was collected from each participant in our study for using his/her data for research studies. All study protocols were approved by the Committee for Human Research at UCSF. The lead-field for each subject was calculated in NUTMEG (Hinkley et al., 2020) using a single-sphere head model and an 8-mm voxel grid corresponding to 5,300 voxels. Each lead-field column was normalized to have a norm of unity. The MEG data were digitally filtered from 1 to 45 Hz to remove artifacts and DC offset. In addition, trials with clear artifacts or visible noise in the MEG sensors that exceeded 10 pT fluctuations were excluded prior to source localization analysis. We experimented on one real MEG auditory evoked fields (AEF) dataset (Cai et al., 2021) to evaluate the performance of our newly introduced brain source imaging method SNC.

### 4.1 Auditory evoked fields data

In this section, we discuss the neural source localization performance of our method structured noise Champagne (SNC) on auditory evoked fields (AEF) in the MEG signal. AEF are often characterized as a function of latency, scalp topography, and the perceptual/cognitive process (Godey et al., 2001). Source localization from auditory evoked fields (AEF) data using MEG measurement is a potential alternative for studying human brain function (Teale et al., 1996). In all experiments with AEF data, the neural response time-series was elicited during passive listening to binaural tones (600 ms duration, carrier frequency of 1 kHz, 40 dB SL). The post-stimulus window in which AEF were analyzed was set to be +50 ms to +150 ms.

**Figure 6:**
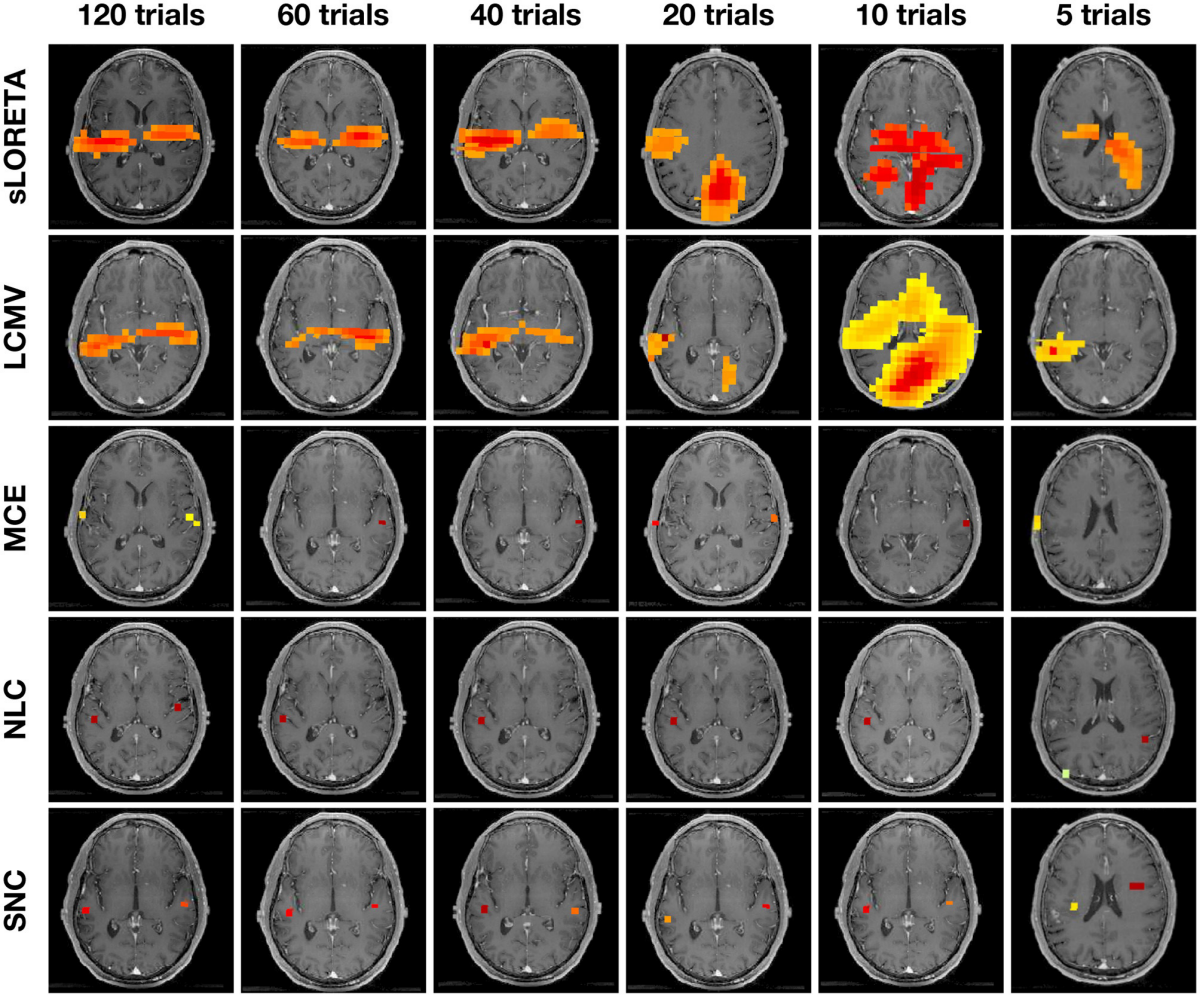
Auditory evoked field (AEF) localization results vs. number of trials from one representative subject using standardized low-resolution brain electromagnetic tomograph (sLORETA), linearly constrained minimum variance (LCMV), minimum current estimate (MCE), noise learning Champagne (NLC), and our method structured noise Champagne (SNC). Note that NLC is able to localize the bilateral auditory activity. In contrast, MCE localization is biased toward the edge of the head. Both sLORETA and LCMV produce some areas of pseudo brain activity.

Figure 6 shows auditory evoked field (AEF) localization results vs. the number of trials from a single representative subject. We compare our result with other methods—sLORETA, LCMV, MCE, and NLC. The power at each voxel around the M100 peak is plotted for each algorithm. SNC is able to localize the expected bilateral brain activation with focal reconstructions under all trial settings. Specifically, the activities localize to Heschl's gyrus in the temporal lobe, which is the characteristic location of the primary auditory cortex. NLC is able to localize the bilateral auditory activity but with shrinkage on one side of the brain activity. The other algorithms do not show robustness compared to SNC. Notice that localization of MCE is biased toward the edge of the head. On the other hand, sLORETA and LCMV produce several areas of pseudo-brain activity. We further note that the LCMV beamformer has a disadvantage in this structured noise scenario due to its well-described weakness for temporally correlated sources as they occur in the auditory cortices for AEFs.

**Figure 7:**
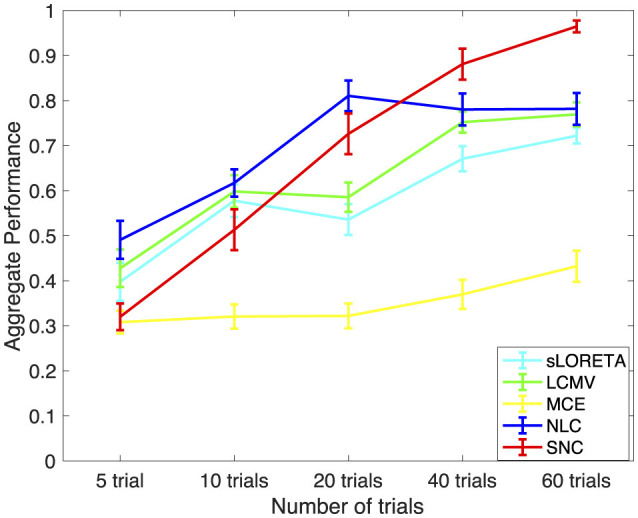
Aggregate performance results vs. number of trials for auditory evoked field (AEF) data using standardized low-resolution brain electromagnetic tomograph (sLORETA), linearly constrained minimum variance (LCMV), minimum current estimate (MCE), noise learning Champagne (NLC), and our method structured noise Champagne (SNC). It is interesting to note that SNC achieves the best aggregated performance after 40 trials. As expected, the performance also improves with the number of trials.

In Figure 7, we present the performance results of sLORETA, LCMV, MCE, NLC, and SNC for AEF localization vs. number of trials for one subject. The error bars in each plot show standard error. Trials are randomly chosen from around 120 trials from each subject, and the number of trials is set in a range from 5 to 60. Each condition is repeated over 30 times for each subject. In this case, we consider the ground truth as the brain activity estimated from approximately 120 trials. In general, increasing the number of trials increases the performance of all algorithms. Notice that all algorithms perform similar when the number of trials is under 10. However, both NLC and SNC work better when the number of trials is above 20. Importantly, when the number of trials increases higher than 40, SNC outperforms all other methods in terms of efficiently localizing the active brain sources.

## 5 Discussion

This study offers an efficient way to estimate contributions to sensors from noise without the need for additional “baseline” or “control” data, while preserving robust reconstruction of complex brain source activity. The underlying data estimation part of our algorithm is based on a principled idea of estimating noise statistics from the model residuals at each iteration of the alternating minimization step of the Champagne algorithm. The key step of the noise learning operation is accomplished by the fact that the residual noise at each iteration exhibits structured precision statistics. The proposed algorithm is readily available to handle a variety of configurations of brain sources under high noise and interference conditions without the need for additional baseline measurements—a requirement that commonly arises in datasets like resting state data analyses. In this context, we note some of the noise/interference reduction strategies—signal space separation (SSS) (Taulu et al., 2005), signal-space projection (SSP) (Uusitalo and Ilmoniemi, 1997), and dual signal subspace projection (DSSP) (Sekihara et al., 2016) for EEG/MEG signals. These methods are primary preprocessing techniques for the EEG/MEG analysis pipeline. In fact, these methods are precisely used to mitigate the noise/interference from the MEG/EEG sensor and external sources. However, none of these are designed to reconstruct the voxel-level time-series. On contrary, our method SNC estimates the MEG/EEG time-series at each voxel of the sensor data.

Exhaustive experimental results demonstrate that the proposed source imaging method offers significant theoretical and empirical advantages over the existing benchmark algorithms when the noise covariance cannot be accurately determined in advance. In simulations, we particularly explored noise learning algorithmic performance for complex source configurations with highly correlated time-courses, and high levels of noise and interference. These simulation results establish the fact that our method outperforms the classical Champagne algorithm (Nagarajan et al., 2007) with an incorrect noise covariance as it achieves higher score of aggregated performance as compared to this and other existing benchmarking methods (Matsuura and Okabe, 1995; Pascual-Marqui et al., 2002; Van Veen et al., 1997; Cai et al., 2021). It is relevant to mention that data-driven approaches, that is, artificial neural networks (ANN)-based inverse solutions, are receiving increasing interest in the literature (Razorenova et al., 2020; Sun et al., 2020; Hecker et al., 2021; Liang et al., 2023). It would be an interesting future extension to explore the scope of our proposed noise learning scheme within these recent artificial intelligence techniques.

To the best of our understanding, the improved performance of this algorithm arises from the efficient the method of estimating the noise statistics via factor analysis of the residual component. Moreover, the proposed structured noise Champagne (SNC) algorithm is found to be robust even when the algorithms are initialized to incorrect noise values. Most importantly, the proposed method is able to robustly localize brain activity with a few trials or even with a single trial in the AEF dataset. This is indeed a significant advancement in electromagnetic brain imaging. We argue that this phenomenon may dramatically cut down the duration of data collection up to 10-fold. This scan-time reduction is particularly important in studies involving children with autism, patients with dementia, or any other subjects who have difficulty tolerating long periods of data collection. In summary, our proposed method offers significant advantages over many existing benchmark algorithms for electromagnetic brain source imaging.

Finally, we would like to discuss some tradeoffs in the current algorithm. Here, we restrict to a diagonal source covariance matrix estimate, which ensures the sparsity of brain sources (Hashemi et al., 2021a), and our convex bounding cost function ensures guaranteed convergence (Wipf et al., 2011; Wipf and Nagarajan, 2009). The structured low-dimensional manifold assumption of the noise covariance helps with accurate estimation with the use of the variational Bayesian factor analysis methods which are robust to smaller data sizes and to the underlying factor dimension specification (Nagarajan et al., 2006, 2007). However, if we want to solve a joint signal and noise estimation problem where both the source and noise covariances are non-diagonal and non-sparse, this problem can become intractable (Hashemi et al., 2020, 2021a). We hope to examine this problem in our future study.

## Data Availability

The raw data supporting the conclusions of this article will be made available by the authors, without undue reservation.

## Appendix

### A1. Proof of Equation 3

Recall that the posterior probability distribution function *p*(*x*_*k*_|*y*_*k*_) is Gaussian which we assumed to be $N\left({\bar{x}}_{k},\Gamma^{-1}\right)$. Furthermore, the exponential part of this Gaussian distribution is given by:

(18)
$$\begin{array}{l} -\frac{1}{2}{\left(x_{k}-{\bar{x}}_{k}\right)}^{T}\Gamma \left(x_{k}-{\bar{x}}_{k}\right) \end{array}$$

$$\begin{array}{l} =-\frac{1}{2}x_{k}^{T}\Gamma x_{k}+x_{k}^{T}\Gamma {\bar{x}}_{k}+C, \end{array}$$

where C stands for the terms that do not contain *x*_*k*_.

We also know that $p\left(y_{k}|x_{k}\right)=N\left(Lx_{k},\Lambda^{-1}\right)$ and $p\left(x_{k}\right)=N\left(0,\Phi^{-1}\right)$. Hence, the posterior probability *p*(*x*_*k*_|*y*_*k*_) can be derived by using Bayes' rule in Equation 2. We see that the exponential part of the posterior probability *p*(*x*_*k*_|*y*_*k*_) takes the form:

(19)
$$\begin{array}{l} -\frac{1}{2}[x_{k}^{T}\Phi x_{k}+{\left(y_{k}-Lx_{k}\right)}^{T}\Lambda \left(y_{k}-Lx_{k}\right)] \end{array}$$

$$\begin{array}{l} =-\frac{1}{2}x_{k}^{T}\left(\Phi +L^{T}\Lambda L\right)x_{k}+x_{k}^{T}L^{T}\Lambda y_{k}+C≃, \end{array}$$

where C≃ again stands for the terms that do not contain *x*_*k*_.

Comparing the quadratic terms of *x*_*k*_ on the right sides of Equations 18, 19:

(20)
$$\begin{array}{l} \Gamma =\Phi +L^{T}\Lambda L. \end{array}$$

Similarly, comparing the linear terms of *x*_*k*_ on the right sides of Equations 18, 19:

(21)
$$\begin{array}{l} \begin{array}{c} {\bar{x}}_{k}=\Gamma^{-1}L^{T}\Lambda y_{k}. \end{array} \end{array}$$

Furthermore, using the matrix inversion formula of Equation (C.92) in Sekihara and Nagarajan (2015), Equation 21 can be rewritten as:

(22)
$$\begin{array}{l} \begin{array}{c} {\bar{x}}_{k}=\Phi^{-1}L^{⊤}{\left(\Lambda^{-1}+L\Phi^{-1}L^{⊤}\right)}^{-1}y_{k}. \end{array} \end{array}$$
